# Cellulose Biosynthesis Inhibitors: Comparative Effect on Bean Cell Cultures

**DOI:** 10.3390/ijms13033685

**Published:** 2012-03-20

**Authors:** Penélope García-Angulo, Ana Alonso-Simón, Antonio Encina, Jesús M. Álvarez, José L. Acebes

**Affiliations:** Department of Agricultural Engineering and Sciences, Plant Physiology Lab, University of León, E-24071, León, Spain; E-Mails: penelope.garcia@unileon.es (P.G.-A.); ana.alonso@unileon.es (A.A.-S.); a.encina@unileon.es (A.E.); jmalvf@unileon.es (J.M.A.)

**Keywords:** AE F150944, cell wall, cell cultures, cellulose biosynthesis-inhibitor (CBI) herbicides, CGA 325′615, compound 1, dichlobenil, *Phaseolus vulgaris* L., quinclorac, triazofenamide

## Abstract

The variety of bioassays developed to evaluate different inhibition responses for cellulose biosynthesis inhibitors makes it difficult to compare the results obtained. This work aims (i) to test a single inhibitory assay for comparing active concentrations of a set of putative cellulose biosynthesis inhibitors and (ii) to characterize their effect on cell wall polysaccharides biosynthesis following a short-term exposure. For the first aim, dose-response curves for inhibition of dry-weight increase following a 30 days exposure of bean callus-cultured cells to these inhibitors were obtained. The compound concentration capable of inhibiting dry weight increase by 50% compared to control (I_50_) ranged from subnanomolar (CGA 325′615) to nanomolar (AE F150944, flupoxam, triazofenamide and oxaziclomefone) and micromolar (dichlobenil, quinclorac and compound 1) concentrations. In order to gain a better understanding of the effect of the putative inhibitors on cell wall polysaccharides biosynthesis, the [^14^C]glucose incorporation into cell wall fractions was determined after a 20 h exposure of cell suspensions to each inhibitor at their I_50_ value. All the inhibitors tested decreased glucose incorporation into cellulose with the exception of quinclorac, which increased it. In some herbicide treatments, reduction in the incorporation into cellulose was accompanied by an increase in the incorporation into other fractions. In order to appreciate the effect of the inhibitors on cell wall partitioning, a cluster and Principal Component Analysis (PCA) based on the relative contribution of [^14^C]glucose incorporation into the different cell wall fractions were performed, and three groups of compounds were identified. The first group included quinclorac, which increased glucose incorporation into cellulose; the second group consisted of compound 1, CGA 325′615, oxaziclomefone and AE F150944, which decreased the relative glucose incorporation into cellulose but increased it into tightly-bound cellulose fractions; and the third group, comprising flupoxam, triazofenamide and dichlobenil, decreased the relative glucose incorporation into cellulose and increased it into a pectin rich fraction.

## 1. Introduction

The presence of a cell wall is a differential characteristic of plant cells, turning this structure as a good candidate for the selection of compounds with herbicide action and presumably lack of action towards animal organisms. Cell walls of growing plant cells (also known as primary cell walls) are complex structures constituted by cellulosic microfibrils embedded in a matrix phase, made of non-cellulosic polysaccharides, with small amounts of proteins, glycoproteins, and proteoglycans, in proportions that depend upon the cell type and its stage of development [1]. These cell wall components are interdependent, and changes in the amount of some of them may trigger modifications in some others (see [2] for a review).

Considering the major cell wall components, noncellulosic or matrix polysaccharides are a group of heteroglycans categorized into two classes: hemicelluloses (mostly composed of neutral sugars forming a linear backbone with short branches, such as xyloglucan, heteroxylans and heteromannans), and pectins (a complex set of galacturonic acid-rich polysaccharides, such as homogalacturonan and rhamnogalacturonans I and II). Matrix polysaccharides are synthesized at Golgi apparatus, transported to the plasma membrane by Golgi-derived vesicles and further incorporated to the cell wall [1].

Cellulose is a β-(1,4) glucan that tends to polymerize into highly ordered structures called microfibrils, synthesized in the outer face of the cell by proteinaceous membrane-bound complexes, constituted by different cellulose synthase (CESA) proteins [3]. Despite the simplicity of cellulose molecule, which is just composed of glucose linked by a single type of bond, the synthesis of this polysaccharide has been shown to be very complex. Cellulose microfibril formation can be divided into three steps: (i) initiation, using UDP-glucose as the donor substrate; (ii) polymerization of glucose into β-(1,4)-glucan chains, and (iii) crystallization of β-(1,4)-glucan chains into a microfibril, a process in which microtubules are implicated [3].

As cellulose is the main component of growing plant cell walls, its biosynthesis has been for decades a desirable target for herbicide action, and a set of putative cellulose biosynthesis-inhibiting compounds (CBIs) has been studied. These inhibitors constitute a group of structurally diverse compounds with different modes of action, although the precise site of action of most CBIs is still unknown (for a recent review see [4]). A selection of CBIs is presented below (Table 1).

Dichlobenil has been used as a CBI for a long time. It has been proposed that it blocks the synthesis of a molecule (sitosterol-β-glucoside) that seems to act as a primer for cellulose biosynthesis [15] and inhibits cellulose biosynthesis by altering the mobility of CESA complexes [16,17] or by preventing the cellulose crystallization through microtubule-mediated effect [18]. Accordingly, different putative dichlobenil targets have been proposed, including a putative regulatory 18 kD protein for β-glucan synthesis [5], CESA1 [19] or CESA5 [20] subunits, and MAP20, a microtubule associated protein in secondary cell wall [21].

AE F150944 acts specifically on organisms which synthesize cellulose via rosettes, through inhibiting crystalline cellulose synthesis. It is thought that its effect is due to the destabilization of plasma membrane rosettes [6].

Flupoxam [7] and triazofenamide [8] are triazole-carboximide herbicides that have been shown to inhibit cellulose biosynthesis and to cause radical changes in cell wall structure and composition [22]. The exact modes of action of the triazole-carboximide herbicides are still unknown.

The mode of action of the thiazolidinone called compound 1 should be similar to that of isoxaben [9] and should differ from the mode of action of triazofenamide, since isoxaben-resistant mutants of *Arabidopsis thaliana* are cross-resistant to compound 1 [9] but sensitive to triazofenamide [10].

The herbicide CGA 325′615 interferes with glucan chain crystallization and causes an accumulation of non-crystalline β-(1,4) glucan [10,23] and also affecting the motility of CESA [24].

Oxaziclomefone reduces the ability of the cell wall to expand [11]. Although possible targets for its action have been studied [12], none of the metabolic processes tested was found to be affected.

There has been some controversy regarding the primary effects of quinclorac. Quinclorac, initially regarded as an “auxin-type” herbicide, has been reported to act as a cell-wall biosynthesis inhibitor in susceptible grasses, since its application inhibits [^14^C]glucose incorporation into cellulose and into a hemicellulose fraction [13]. Nevertheless, no further evidence that quinclorac inhibits either cellulose or cell wall polysaccharide biosynthesis has been found [14,25].

In addition to these, other compounds such as isoxaben and thaxthomin have been widely used, at least since a decade, as experimental CBIs [4]. In the last few years, some other compounds with different activities, such as growth retardants or anti-microtubule agents, have also been proposed to indirectly inhibit cellulose biosynthesis (for a recent review see [4]). These compounds include ancymidol [26], the coumarin derivative, morlin [27], cobtorin [28,29], triaziflam [30], indaziflam [31] and MBTU (1-α-methylbenzyl-3-*p*-tolylurea) [32,33].

The abovementioned inhibitors were reported to carry out different actions on the cell wall and to require a different range of active concentrations. However, the variety of bioassays tested for evaluating different inhibition responses for each of these compounds makes it difficult to compare the results obtained. Consequently, comparison of their activities requires a single bioassay which standardizes inhibition parameters. In addition, the varying half-inhibition concentration (I_50_) values may be due to the different species used in the various studies carried out. Accordingly, the aims of the present work were: (i) to test a single inhibitory assay for comparing the active concentrations of eight compounds (AE F150944, CGA 325′615, compound 1, dichobenil, flupoxam, oxaziclomefone, quinclorac, triazofenamide) reported to alter cell wall formation by inhibiting cellulose biosynthesis and (ii) to identify the short-term effect of these compounds on cell wall composition and carbon flow towards the cell wall polysaccharides.

We have reported elsewhere that bean calluses constitute a suitable plant material for investigating the effect of some putative CBI herbicides, such as isoxaben [34], dichlobenil [35] and quinclorac [25]. Therefore, for the first aim, dose-response curves for dry-weight (DWt) increase inhibition were obtained after long exposure (30 days) of bean calluses to each inhibitor. For the second aim, the effect of the brief exposure (20 h) of cell suspensions growing at the exponential growth phase to the inhibitors on [^14^C]glucose uptake into cell wall fractions was determined, and principal component analysis and cluster analysis were applied to the results obtained.

## 2. Results and Discussion

### 2.1. Inhibition of Callus Growth

**Figure 1:**
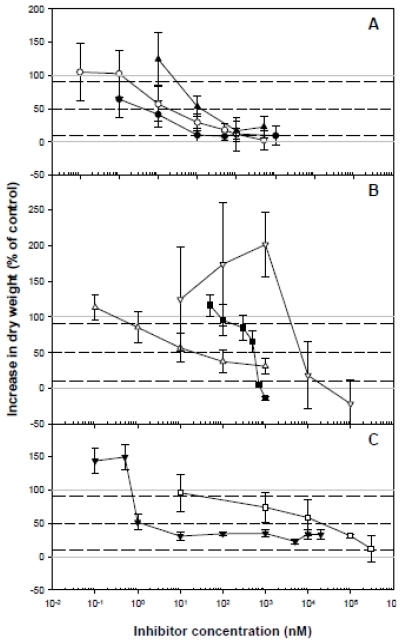
Dose-response curves for several putative CBIs on bean calluses growth. Calluses growth was calculated as the percentage increase in dry weight relative to the untreated calluses after 30 d culture. **A**: (●) CGA 325′615, (▴) triazofenamide, (□) compound 1; **B**: (▵) oxaziclomefone, (■) dichlobenil, (▿) quinclorac; **C**: (○) flupoxam, (▾) AE F150944. Values are means ± SD of 8 measurements. Dotted lines were included in order to estimate I_10_, I_50_ and I_90_ values. Solid grey lines indicate the 100% and 0% of growth.

Comparison of active concentrations requires a single bioassay which standardizes the inhibition parameters. We chose a bioassay based on the inhibition of DWt gain in callus-cultured cells since this has previously produced good results in the study of the action of isoxaben [34], dichlobenil [35] and quinclorac [25]. The effect of increasing concentrations of the compounds tested here on DWt gain in bean calluses after 30 days of culture is shown in Figure 1, and the derived inhibition parameters are shown in Table 2.

The most active inhibitor was CGA 325′615, showing an I_50_ value in the subnanomolar range, in contrast to quinclorac, dichlobenil and compound 1, with I_50_ values in the micromolar range. Overall, the I_50_ values reported here do not differ notably from those obtained by other research groups, although the broad range of bioassays developed for each of these inhibitors makes it difficult to compare the results obtained. The reported variation in half-inhibition concentration values (I_50_) may also be due to the different species used. The root growth of several dicots is 50% inhibited in the nanomolar range by triazofenamide (I_50_: 39 nM) [8], flupoxam (I_50_: 6 nM) [7] and dichlobenil (I_50_: 400 nM) [8], whilst quinclorac reduces root growth in the micromolar range (I_50_: 5 μM) [13]. The I_50_ values for the different inhibitory responses to compound 1 [9], CGA 325′615 [10], AE F150944 [6] and oxaziclomefone [11] also lie within the nanomolar range for dicot plants.

Quinclorac, AE F150944, and to a lesser extent triazofenamide (Figure 1), were the only herbicides to cause a significant increase in DWt after 30 days of callus culture at low concentrations (below their respective I_10_). This biphasic dose-response phenomenon, characterized by low-dose stimulation and high-dose inhibition, called hormesis, has been reported in a large number and wide range of toxicological studies, including those concerning the effects of herbicides on plants [36–38]. The phenomenon is not well understood, but has been attributed to low levels of potential toxins or stress, which first cause a disruption to, and then an overcompensation of, the mechanisms controlling the measurable response. However, the stimulatory action of quinclorac could be associated with an auxin-dependent regulation of hydrolytic enzymes or with the induction of ethylene, cyanide, or reactive oxygen species [39–41].

The I_90_/I_10_ ratio (Table 2) indicates the amplitude of the active concentration range, which is also an important parameter for establishing a reference frame with which subsequent inhibitors can be compared. AE F150944 had the highest quotient, whereas quinclorac and dichlobenil showed the lowest one.

Most of the inhibitors, at their respective I_50_ (and more notably at their I_90_), enhanced the DWt/fresh weight (FWt) ratio with respect to untreated calluses. This effect could be related to extensive cell death due to an alteration of callus growth caused by the presence of the inhibitor: cell walls from dead cells remain bound to the calluses, and contribute to a higher proportion of DWt/FWt ratio.

Cellulose content was evaluated in cell walls from calluses cultivated in the presence of a herbicide after a 30 days treatment with a concentration equal to the I_50_ value in order to establish whether the putative inhibitors were able to reduce this content (Table 2). The amount of cellulose accounted for approximately 210 μg per mg of crude cell wall DWt in non-treated calluses, and none of the herbicide treatments produced a significant reduction in the amount of cellulose, which in contrast increased by 45%, 27% and 18% after treatment with compound 1, triazofenamide and AEF150944, respectively. These results show that there may be a considerable difference between short time responses and long time responses, in which changes in cell wall composition may trigger signalling pathways providing a sensing mechanism through which cell responses can be co-ordinated or altered appropriately to remodel cell wall composition in order to cope with stress factors [42].

### 2.2. Uptake of [^14^C]Glucose after Short-Term Exposure to the Inhibitors

**Figure 2:**
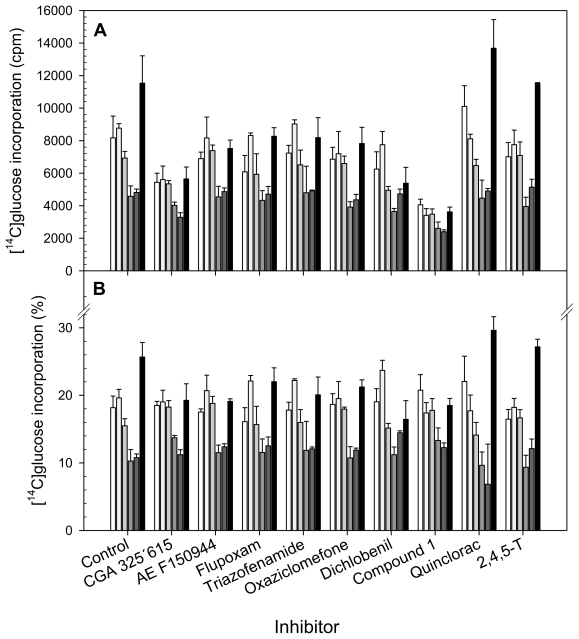
[^14^C]Glc incorporation into cell wall fractions: CDTA (□), Carbonate ( 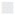), KOH ( 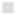), Sn-CR ( 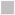), ANW ( 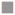) and α-Cellulose (■) from cell suspensions untreated (Control) or treated for 20 h with different CBIs at their I_50_ concentration (see Table 2). Data are expressed as total cpm incorporated into each fraction (**A**) and as percentages of incorporation in each fraction regarding to total incorporation into the cell wall (**B**). Values are means ± SD of three technical replicates.

**Figure 3:**
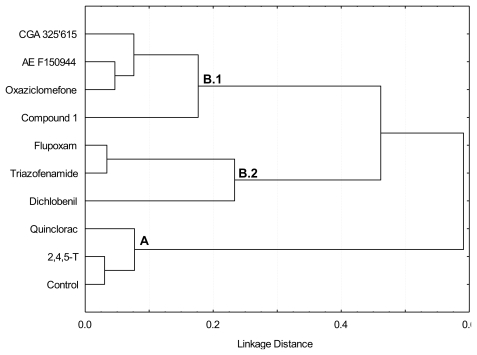
Dendrogram obtained by cluster analysis of relative [^14^C]glucose incorporation into cell wall fractions from cell suspensions cultured for 20 h in the presence of putative CBIs at the I_50_ concentration. **A**, **B.1** and **B.2** are the branches discussed in the text.

**Figure 4:**
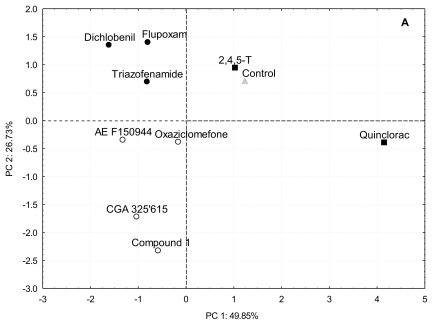

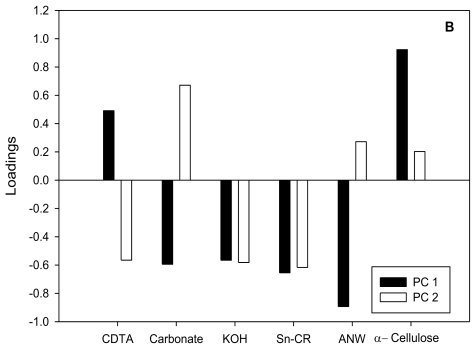
(**A**) Principal Component Analysis of relative [^14^C]glucose incorporation into cell wall fractions of cell suspensions cultured for 20 h in the presence of putative CBIs at the I_50_ concentration. The inhibitors were grouped in the same way that in cluster analysis: cluster A (■ and control ▵), cluster B.1 (○) and cluster B.2 (●); (**B**) Loadings for principal component 1 (□) and 2 (■).

In order to characterize the effect of the inhibitors on cell wall biosynthesis, and to avoid collateral effects in long-term exposures, the effect of a brief exposure (20 h) to the I_50_ concentration of each inhibitor on [^14^C]glucose uptake into cell wall fractions was determined (Figure 2A,B). The cell wall polysaccharides were sequentially extracted by treatment with CDTA and carbonate, which mainly extract pectins, and then by treatment with KOH, that solubilized hemicelluloses. The polysaccharides tightly bound to cellulose were collected in Supernatant Cellulose Residue (Sn-CR) and Acetic acid/Nitric acid/Water (ANW) fractions. Further, to better understanding the differences between inhibitors, a cluster (Figure 3) and Principal Component Analysis (Figure 4) on the basis of [^14^C]glucose incorporation into cell wall fractions were obtained. Since quinclorac has been described both as CBI and as an auxinic herbicide, the well-known auxin 2,4,5-trichlorophenoxyacetic acid (2,4,5-T) was included in this study in order to compare its effects with those from quinclorac, trying to clarify the action of this compound.

The pattern of [^14^C]glucose uptake into the different cell compartments was similar for all the inhibitors tested and for the control. [^14^C]glucose incorporation into the cell wall was low in comparison to its incorporation into the cytosol, which was the main sink for [^14^C]glucose (data not shown). With regard to [^14^C]glucose incorporation into the cell wall fractions of untreated cells (Figure 2A), the highest incorporation was observed for α-cellulose, whereas the lowest labeling was found for the Sn-CR and ANW fractions. [^14^C]glucose incorporation into pectins from both the CDTA and carbonate fractions was slightly higher than into hemicelluloses. This incorporation pattern was very consistent in all the incorporation experiments (Figure 2A). [^14^C]glucose incorporation into cell wall was reduced by all CBI-treatments except by quinclorac and 2,4,5-T. Direct comparison among treatments was difficult. So, in order to deepen understanding of the CBIs in the cell wall compared with the control and to identify trends on carbon flow towards the cell wall polysaccharides, percentages of incorporation into each fraction regarding to total incorporation into the cell wall were calculated (Figure 2B). Except for the cases of quinclorac and 2,4,5-T, treatments with the inhibitors reduced the [^14^C]glucose incorporation into α-cellulose (between 14 and 36%), and increased it for the tightly bound cellulose fractions (Figure 2B). This fact demonstrates that they effectively are able to affect cellulose biosynthesis, and illustrates that a difference among short and long term responses exists. By contrast, quinclorac increased [^14^C]glucose incorporation into α-cellulose and decreased it for the ANW fraction, and the synthetic auxin (2,4,5-T) showed similar effects. Treatment with compound 1 or quinclorac increased incorporation into the CDTA-extracted pectins and decreased it into polysaccharides extracted in carbonate fraction, whilst flupoxam had the opposite effect and dichlobenil only increased incorporation into the carbonate fraction. Finally, the incorporation of [^14^C]glucose into KOH-soluble hemicelluloses increased after exposure to CGA 325′615, AE F150944, compound **1** and oxaziclomefone.

Two initial groups were identified in the dendrogram obtained by cluster analysis with respect to the pattern of [^14^C]glucose incorporation into α-cellulose (Figure 3). The first group was formed by those treatments (control, 2,4,5-T and quinclorac) in which the incorporation of [^14^C]glucose into the α-cellulose fraction were higher than 25% of the total incorporation into cell wall; these were located in branch A of the dendrogram. The second group included those treatments which inhibited incorporation of [^14^C]glucose into α-cellulose respect to the control, and were located in branch B of the dendrogram: flupoxam, dichlobenil, triazofenamide, compound 1, CGA 325′615, oxaziclomefone and AE F150944. The compounds in the second group were further separated into two subgroups according to the pattern of [^14^C]glucose incorporation into pectin or hemicellulose fractions. Those treatments that increased in percentage incorporation of [^14^C]glucose into KOH-extracted hemicelluloses and polysaccharides tightly bound to the cellulose (Sn-CR fraction) were located in sub-branch B.1 (CGA 325′615, AE F150944, oxaziclomefone and compound **1**). However, flupoxam, triazofenamide and dichlobenil diverted carbon flux towards pectic polysaccharides solubilized with carbonate, and also towards polysaccharides extracted with ANW (and/or Sn-CR fraction to a lesser extent), and were located in sub-branch B.2.

Results obtained from relative [^14^C]glucose incorporation into cell wall fractions were further analyzed by Principal Component Analysis (PCA) (Figure 3A,B), a multivariate analysis that orders variables regarding gradients along Principal Components (PCs). Almost 80% of total variance was explained by PC1 and PC2. According to factor loadings (Figure 3B), PC1 (approx. 50% of variance explained) had a strong positive correlation with α-cellulose and a negative correlation with ANW. Therefore, the analysis located the inhibitors in opposite sides of PC1, depending on their patterns of glucose incorporation into α-cellulose. Those compounds able to inhibit the incorporation of [^14^C]glucose into cellulose (flupoxam, dichlobenil, triazofenamide, compound 1, CGA 325′615, oxaziclomefone and AE F150944) were located on the negative side of PC1 (Figure 3A). On the other hand, those compounds that did not affect or increased incorporation into α-cellulose, such as quinclorac and 2,4,5-T, were located towards the positive side of PC1, grouping with untreated cells. Comparing this result with the dendrogram (Figure 2), there is a perfect match of A and B branches with the groups obtained by PCA. Thus, these two different multivariate analyses detect the same blocks of glucose incorporation patterns, corroborating the differences between these groups of inhibitors.

A negative correlation was found between relative [^14^C]glucose incorporation into α-cellulose and into polysaccharides tightly-bound to cellulose. Thus, those compounds that reduced [^14^C]glucose incorporation into α-cellulose also increased incorporation into the ANW fractions (and/or Sn-CR fraction in a lesser extent). We propose that this result is a consequence of the interference in the crystallization of β-(1,4) glucan chains into a microfibril rather than in the polymerization of glucose into β-(1,4) glucan chains. Several results would agree with this explanation. (i) Whenever treatment with an inhibitor caused an increase of [^14^C]glucose uptake into the Sn-CR fraction, this fraction was subjected to endoglucanase-digestion, followed by thin layer chromatography of the released products. In all cases, a peak at Rf 0, plus an additional broad peak that co-migrated with cello-oligosaccharides such as cellotriose or cellobiose, were obtained (data not shown), suggesting the presence of a soluble β-(1,4) glucan; (ii) In dichlobenil-habituated cell walls, the characteristic reduction in α-cellulose was paralleled by a notable increase in polysaccharides tightly-bound to cellulose, which were also enriched in glucose, presumably derived from a non-crystalline β-(1,4) glucan [43]. In accordance with this explanation it is plausible that the inhibitors in the second group cause the same effect; (iii) CGA 325′615 has been reported to decrease cellulose biosynthesis and to cause a concomitant accumulation of non-crystalline β-(1,4) glucan by disrupting the crystallization of β-glucan chains into a microfibril [10]; (iv) AE F150944 specifically inhibits crystalline cellulose synthesis by destabilizing plasma membrane rosettes in *Zinnia elegans* [6] and this destabilization might cause a deviation in [^14^C]glucose incorporation from the α-cellulose to the Sn-CR fraction.

The reduction of relative [^14^C]glucose incorporation into α-cellulose would generate a surplus of glucose to be diverted towards matrix polysaccharides. According to our results, the carbon diversion into matrix polysaccharides would follow two alternative routes. PC2 and cluster analysis further separate CBIs (branch B) into two groups. Flupoxam, triazofenamide and dichlobenil seem to divert carbon flux towards carbonate-extracted pectins (positive side of PC2; branch B.2). On the other hand, CGA 325′615, AE F150944, oxaziclomefone and compound **1** would appear to increase the incorporation into KOH hemicelluloses and/or CDTA extracted pectins to a lesser extent (negative side of PC2; branch B.1). These two putative pathways of carbon reflux could reflect two different sets of down-stream effects on cellulose inhibition (*i.e.*, inhibition of hemicelluloses synthesis, increase in pectic polysaccharide synthesis), although this does not necessarily mean that all inhibitors of the same group have exactly the same mode of action.

There is controversy regarding the primary effect of quinclorac. It has previously been reported that quinclorac inhibits cell wall biosynthesis in susceptible grasses [13]. However the effect of quinclorac as a CBI has since been questioned [14,25], and no further evidence that this compound inhibits cellulose biosynthesis in roots of susceptible grasses, either directly or indirectly, has appeared. In order to elucidate the mode of action of quinclorac, here we also studied the effect of the auxin 2,4,5-T on the incorporation of glucose into cell wall polysaccharides, as quinclorac was initially regarded an auxin-type herbicide. Our data were not consistent with an inhibition of cellulose biosynthesis since quinclorac increased [^14^C]glucose incorporation into the cellulosic fraction, and was grouped together with 2,4,5-T both in dendrogram and PCA. Considering all these results, we would suggest that quinclorac acts as an auxin and that the modification in the pattern of glucose incorporation could be a side effect of its mode of action. In this sense, it has been reported that tolerance to quinclorac occurs through a target site-based mechanism involving stimulation of ACC synthesis and a higher β-cyanoalanine synthase activity [44]. Moreover, we have recently observed that long-term modifications of the cell wall caused by the habituation of bean cell cultures to quinclorac did not resemble those of bean cells habituated to the well-known CBIs dichlobenil or isoxaben [25]. In sum, quinclorac could be classified as a drug that display a dual effect, acting as CBI in some cases (depending on the species or their concentration), and as auxin herbicide in others [4].

## 3. Experimental Section

### 3.1. Cell Cultures

Bean calluses were obtained from seedling leaves, as previously described [35]. Calluses were cultured on Murashige and Skoog medium [45] containing 8 g L^−1^ agar and 10 μM 2,4-D. Calluses were removed from the explants and routinely subcultured for 30 days on identical medium. Cell suspensions were obtained from calluses cultured in liquid Murashige and Skoog medium containing 5 μM 2,4-D and shaken on a rotary shaker.

### 3.2. Effect of Inhibitors on Calluses Growth

Calluses (0.5–0.7 g) were subjected to 30 days incubation in growth medium supplemented with a range of inhibitor concentrations. The inhibitors were dissolved in ethanol, except quinclorac, which was dissolved in DMSO. The final concentration of DMSO or ethanol did not affect calluses growth. Cultured cells were then weighed (FWt) and dried at 60 °C until constant weight was achieved (DWt). Growth was expressed as the relative increase in FWt and DWt. I_10_, I_50_ and I_90_ values were calculated as the concentration of compound able to inhibit the increase in DWt by 10%, 50% and 90% respectively.

### 3.3. Cellulose Analysis

Cellulose was quantified in crude cell walls with the Updegraff method [46] using the hydrolytic conditions described by Saeman *et al.* [47], and the glucose released was determined with the anthrone assay [48]. Anova followed by Tukey test (*p* < 0.05) was used for variance analysis.

### 3.4. [^14^C]Glucose Uptake

The uptake of [^14^C]glucose was carried out following the method described previously [49] with some modifications. Cell suspensions were collected in the exponential growth phase (15 days after subculture) and were washed with glucose-free culture medium using a glass fibre filter. Cells were then resuspended in 20 mL of the same medium (uniformly aliquoted as 30% settled cell volume, about 1.33 g FW cells) containing the inhibitor at a final concentration equal to the I_50_ value (see Table 1) (except for 2,4,5-trichlorophenoxyacetic acid, which was used at a final concentration of 7 μM) and incubated for 1 h at 25 °C. Then, [^14^C]glucose was added at a final concentration of 10 μM and the cell suspensions were shaken at 130 rpm at 25 °C.

For each incorporation experiment, three analytical replicates were carried out. At 20 h after the addition of the labelled substrate, cells were transferred to three Poly-Prep columns (BioRad) (4 mL × column) and all subsequent washes and fractionations were carried out in these tubes. First, cells were washed with 70% ethanol (×3) and 0.01 M phosphate buffer, pH 7.0 at room temperature for 24 h. Starch was removed by treatment with 2.5 mU mL^−1^ of α-amylase (hog pancreas type VI) in the same buffer for 24 h at room temperature. The suspension was then removed and the pellet was washed with phenol/acetic acid/water (2/2/1, v/v/v) over 8 h and with 70% ethanol (×2) and acetone (×3), after which it was air-dried. The combination of ethanol, α-amylase and phenol/acetic/water extractions were considered as the cytosolic fraction. Cell walls were fractionated as described below.

### 3.5. Cell Wall Fractionation

Cell wall fractionation was performed according to a slightly modified version of the method described by Coimbra *et al.* [50]. Dry walls were extracted for 8 h at room temperature with 50 mM *t*-1,2-diaminecyclohexane-N,N,N′,N′-tetraacetic acid (CDTA) sodium salt at pH 6.5, and then washed with distilled water. Solubilized compounds and washing were combined and constituted the CDTA fraction. Fifty mM Na_2_CO_3_ plus 20 mM NaBH_4_ was added to the pellet and the suspension was kept for 18 h at room temperature and then washed with distilled water (carbonate fraction). Following this, hemicelluloses were solubilised by incubating the pellet for 18 h with 4 M KOH plus 20 mM NaBH_4_ and washed with distilled water (KOH fraction). The residue of the KOH extraction was suspended in water and adjusted to pH 5.0 with acetic acid. The supernatant was collected and referred to as Supernatant Cellulose Residue (Sn-CR fraction). The residue was hydrolysed for 2.5 h with a mixture of Acetic acid/Nitric acid/Water (8/1/2 v/v/v), and the solution was designated the ANW fraction. Finally, the residue was washed with distilled water and air-dried (α-cellulose fraction). Aliquots were collected during cell wall isolation and fractionation, and mixed with liquid scintillation solution. Radioactivity was determined on a Beckman LS6000TA scintillation counter. Results were expressed as total cpm incorporated into each fraction and as a percentage of incorporation into each cell wall fraction regarding total incorporation in the cell wall. Values are means ± SD of three analytical replicates.

Cluster analysis of [^14^C]glucose incorporation into cell wall fractions (expressed as percentages; Figure 2B) was performed using the Ward method and the Pearson coefficient was selected as the distance measurement. PCA of [^14^C]glucose incorporation into cell wall fractions was performed using a maximum of five principal components. All statistical analyses were carried out using the Statistica 6.0 software package [51].

## 4. Conclusions

To sum up, the results obtained show that CBIs form a heterogeneous group with different inhibition parameters and range of active concentrations. These compounds affect the cell wall in different ways depending on whether the exposure period is short (20 h) or long (30 days), and can be clustered into several subgroups displaying different modes of action and affecting distinct stages of the cellulose biosynthesis process. Thus, [^14^C]glucose uptake into cell wall fractions showed that flupoxam, dichlobenil, triazofenamide, compound **1**, CGA 325′615 and AE F150944 could act by altering β-glucan chain crystallization rather than by inhibiting glucose polymerization. However, two subgroups can be identified in this group: the first three inhibitors diverted the carbon flux into carbonate-extracted pectic polysaccharides, whereas the last three diverted it into hemicelluloses. The action of quinclorac on the cell wall could be associated with its auxin nature. Further research is necessary in order to establish the exact mechanism of action of these inhibitors.

## Figures and Tables

**Table 1:** Accepted chemical names of selected CBIs and references about them.

CBI	Chemical Name	References
Dichlobenil	2,6-dichlorobenzonitrile	[5]
AE F150944	*N*2-(1-ethyl-3-phenylpropyl)-6-(1-fluoro-1-methylethyl)-1,3,5-triazine-2,4-diamine	[6]
Flupoxam	1-[4-chloro-3-[(2,2,3,3,3-pentafluoropropoxymethyl) phenyl]-5-phenyl-1*H*-1,2,4-triazole-3-carboximide	[7]
Triazofenamide	1-(3-methyl phenyl)-5-phenyl-1*H*-1,2,4-3 triazole-3-carboximide	[8]
Compound 1	5-*tert*-butyl-carbamoyloxyl-3-(3-trifluoromethyl) phenyl-4-thiazolidinone	[9]
CGA 325′615	1-cyclohexyl-5-(2,3,4,5,6-pentafluorophenoxy)-1 λ4,2,4,6-thiatriazin-3-amine	[10]
Oxaziclomefone	3-(1-(3,5-dichlorophenyl)-1-methylethyl)-3,4-dihydro-6-methyl-5-phenyl-2*H*-1,3-oxazin-4-one	[11,12]
Quinclorac	3,7-dichloro-8-quinoline carboxylic acid	[13,14]

**Table 2:** Inhibition parameters of several putative CBIs on bean callus growth. I_10_, I_50_ and I_90_ values were calculated as the concentration of inhibitor able to inhibit the increase in dry weigth (DWt) by 10%, 50% and 90% respectively. The active concentration range was expressed as the ratio I_90_/I_10_. Dry weigth (DWt)/fresh weight (FWt) ratio was estimated at I_50_ and I_90_ concentrations. The DWt/FWt from callus cultures growing in the absence of any inhibitor was 0.046. Cellulose content of cell walls isolated from calluses was measured after 30 days culture in the presence of the inhibitors at I_50_ concentration. Cellulose content in control was 212.6 ± 23.6 μg mg^−1^ CW. Values are means ± SD of 9 measurements.

	Inhibition Parameters				DWt/FWt		
		
CBI	I_10_	I_50_	I_90_	Active Concentration Range (I_90_/I_10_)	DWt/FWt (I_50_)	DWt/FWt (I_90_)	Cellulose (μg mg^−1^ CW)
CGA 325′615	<0.1 nM	0.5 nM	10 nM	100	0.048	0.052	241.7 ± 33.3
AE F150944	0.8 nM	1 nM	>20 mM	~25000	0.053	~0.064	277.2 ± 18.7 *
Flupoxam	0.2 nM	2 nM	400 nM	2000	0.048	0.058	216.3 ± 5.5
Triazofenamide	4 nM	15 nM	100 nM	25	0.047	0.046	258.5 ± 29.3 *
Oxaziclomefone	0.6 nM	30 nM	>1 μM	~1667	0.048	~0.052	229 ± 22.7
Dichlobenil	0.2 μM	0.5 μM	1 μM	5	0.032	0.048	220.4 ± 18.4
Quinclorac	4 μM	10 μM	20 μM	5	0.050	0.053	247.9 ± 4.1
Compound 1	20 nM	20 μM	200 μM	10000	0.055	0.060	328.5 ± 37.2 *

* Values statistically different of control by Tukey test *p* < 0.05.
